# Clinical study of XiangShaLiuJunZi decoction combined with S-1 as maintenance therapy for stage III or IV gastric carcinoma and colorectal carcinoma

**DOI:** 10.1097/MD.0000000000020081

**Published:** 2020-05-08

**Authors:** Xiao-Cui Hong, Qi-Lian Liang, Xing-Bo Luo, Ke-Hui Hu, Hai-Xia Yang, Wen-ting Ou, Hui-Jie Zhang

**Affiliations:** Oncology Center, Affiliated Hospital of Guangdong Medical University, Zhanjiang, China.

**Keywords:** colorectal carcinoma(CRC), gastric carcinoma(GC), maintenance therapy, randomized controlled trial(RCT), S-1, xiangshaliujunzi decoction(XSLJZD)

## Abstract

**Introduction:**

S-1, a new oral fluorouracil chemotherapeutical drug, has been increasingly used in clinical maintenance after first-line chemotherapy for stage III or IV gastric carcinoma (GC) and colorectal carcinoma (CRC) for its own advantages. XiangshaLiujunzi Decoction (XSLJZD), a classic traditional Chinese medicine (TCM) formula with effects of alleviating the adverse reactions of chemotherapy and improving the quality of life of cancer patients has been gradually confirmed, with no more reports about the maintenance therapy mode of combination of chemotherapeutic drugs and TCM. We designed the study of XSLJZD combined with S-1 in the maintenance therapy of Stage III or IV GC and CRC, and hoped that this research program will go further and comprehensively evaluate its efficacy and safety.

**Objectives:**

The aim of this study was to determine the efficacy and safety of XSLJZD combined with S-1 in the maintenance therapy of stage III or IV GC and CRC.

**Methods:**

This study is an open, single-center, randomized study. Patients with stage III or stage IV GC and CRC will be randomized (1:1) into S-1group, S-1 combined with XSLJZD group for 5 years of maintenance therapy. The primary endpoint was progression-free survival, and secondary end point was overall survival and Quality of Life Assessment (QOLA), which include an improvement in symptoms before and after treatment, Karnofsky Performance Status, and adverse events assessment.

**Discussion:**

This study will provide meaningful clinical information about the combination of chemotherapeutic drugs S-1 with TCM in the maintenance therapy of stage III or IV GC and CRC.

**Trial registration:**

Chinese Clinical Trial Registry: ChiCTR-INR-16008575.

## Abstract 2

Supplemental Digital Content is available in the text

## 1 Introduction

Gastric carcinoma (GC) is the fifth malignancy and the third cause of cancer death worldwide, and colorectal carcinoma (CRC) is the third most common fatal cancer worldwide.^[1,2]^ The curative treatment for non-early GC and CRC is mainly surgical, and the treatment for advanced GC and CRC is a multimodal treatment model based on chemotherapy. Due to the several phase III studies showing the treatment benefits of neoadjuvant and adjuvant chemotherapy protocols, there have been major advances in the multimodal treatment of GC and CRC.^[3–5]^ Although the total effective rate of first-line and second-line chemotherapy is better than before, the prognosis of GC and CRC still needs to be improved.

Maintenance therapy refers to the mode that patients with advanced cancer who have been given by drug treatment and have their condition under control will stop the original treatment plan and switch to other plans for the next treatment until the disease progresses. In some studies on maintenance therapy for GC, patients without progression after firstline chemotherapy that include docetaxel, cisplatin and XELOX were randomly assigned to receive capecitabine maintenance. The results showed that the median progression-free survival (PFS) and median overall survival time (OS) of the patients receiving maintenance therapy were significantly better than that of the group without maintenance therapy, and grade 3/4 adverse events (AEs) and hematological toxicity were less common during maintenance therapy.^[6,7]^ The same result has also been found in GC studies where the targeted drug trastuzumab was selected as maintenance therapy.^[8]^ These studies suggest that maintenance therapy may benefit patients with advanced GC after chemotherapy, but more research is needed in this regard.

The maintenance therapy of advanced CRC is more mature than that of GC. During the past decade, capecitabine or capecitabine combined with bevacizumab has been repeatedly used in maintenance therapy after first-line chemotherapy studies for advanced CRC, and the results showed that the PFS and OS of the maintenance group were improved compared with the control group.^[9–11]^ The evidence shows that the use of fluorouracil or targeted drugs in the maintenance therapy of advanced CRC can benefit the survival of patients, which preliminarily proves that the maintenance model is feasible in the treatment of patients with advanced CRC.

Capecitabine has been widely used as an oral fluorouracil chemotherapeutical drug in chemotherapy for GC and CRC, and the new oral fluoropyrimidine S-1 was developed by Japan; studies have shown that it may be more suitable for Asians. It is a compound preparation whose main components are tegafur, gimeracil, and potassium oxonate, tegafur acts as a prodrug of 5-fluorouracil (5-FU), gimeracil maintains the serum concentration of 5-FU, and potassium oxonate inhibits the phosphorylation of 5-FU in the gastrointestinal tract to potentially decrease serious gastrointestinal toxicities. At present, S-1 is widely used as the adjuvant and palliative chemotherapy for GC and CRC.^[12–16]^

Now many studies have shown that the application of traditional Chinese medicine (TCM) can significantly reduce the adverse reactions caused by chemotherapy, improve postoperative maintenance, prevent recurrence and metastasis, enhance patient quality of life, and prolong survival.^[17–20]^ XiangshaLiujunzi Decoction (XSLJZD)^[21–24]^ is a classic TCM formula containing 8 commonly used herbs (Radix codonopsis, Rhizoma atractylodis macrocephalae, Radix glycyrrhizae, Poria, Pericarpium citri reticulatae, Pinellia tuber, Radix aucklandiae, and Fructus amomi). With effects of invigorating the spleen and replenishing qi, XSLJZD has long been used to treat gastrointestinal discomfort in clinical practice in China, including those caused by chemotherapy. The use of TCM for tumor maintenance therapy is still inception, but it is gratifying that these preliminary exploratory studies have given some hope to this new clinical treatment model. Tong et al used the modified XSLJZD combined XELOX scheme in treatment of the advanced GC, and found that the modified XSLJZD alleviates the adverse reactions of XELOX chemotherapy and improves the quality of life and immunity as well as the therapy effects in the patients with advanced GC.^[25]^

In recent years, the maintenance therapy mode of lung cancer has become more mature, and there is still room for gastrointestinal tumors to develop. Besides, there has no report on maintenance therapy mode of combination of chemotherapeutic drugs with TCM. Therefore, this study will aim to combine the research foundation of the preliminary small-scale exploratory trials to carry out a randomized, controlled trial for further evaluation of the efficacy and safety S-1 combined with XSLJZD in the maintenance therapy of stage III or IV GC and CRC.

## 2 Methods and analysis

### 2.1 Study design

**Figure 1:**
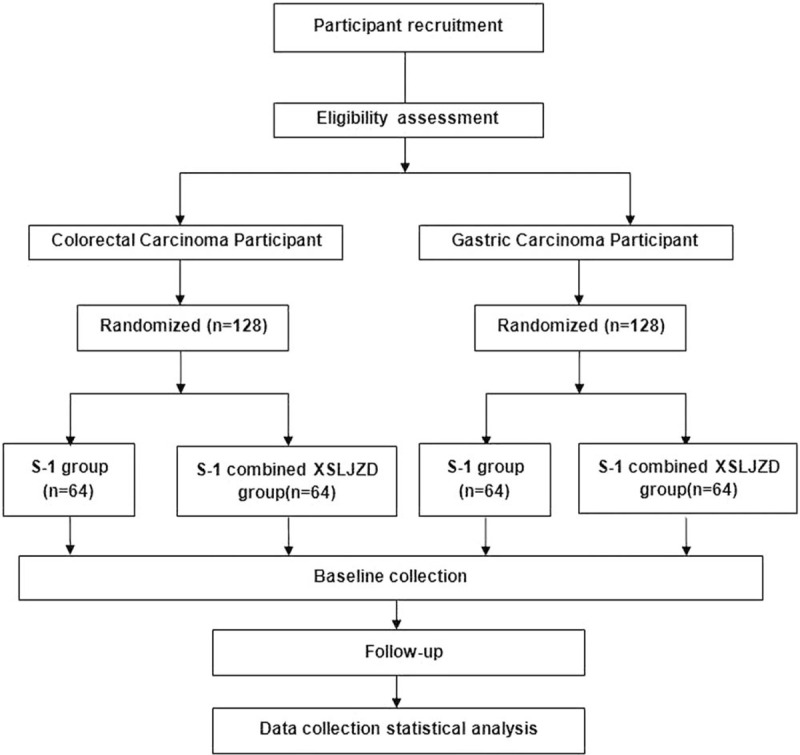
The flowchart of the study.

This study is an open, single-center, randomized controlled trial (RCT), wherein 256 patients with stage III or IV GC and CRC (128 patients of each carcinoma type) who met the inclusion criteria will be randomly assigned to 2 groups, receiving S-1 or S-1 combined with XSLJZD for 5 years. The flowchart of the study is presented in Figure 1.

The reporting of the protocol follows the Standard Protocol Items: Recommendations for Interventional Trials 2013 (SPIRIT2013) guidelines.^[26,27]^

### 2.2 Recruitment

The study was carried out at the Oncology Center of the Affiliated Hospital of Guangdong Medical University. The participants were all inpatients of the Oncology Center of Affiliated Hospital of Guangdong Medical University, mainly recruited through Internet advertisements and hospital posters. The patients interested in participating in the study will be evaluated by clinicians to determine their qualifications. The clinician will inform the patient of the detailed study objectives, process, and potential benefits and risks. All participants will sign the informed consent before screening.

### 2.3 Inclusion criteria

Patients will be enrolled in this trial if they meet the following criteria: there must be an informed consent form signed by the patient himself or by the witness; TNM III-IV GC or CRC; controlled by chemotherapy (CR/PR/SD); age 18 to 75 years (≥18 years’ old, ≤75 years’ old), expected survival period is >3 months; KPS ≥60^[28]^; follow the Response Evaluation Criteria In Solid Tumors (RECIST 1.1),^[29]^ at least 1 measurable lesion; routine blood test: hemoglobin ≥90 g/L; neutrophil count ≥1.5 × 10^9^ cells/L; platelet count ≥100 × 10^9^ cells/L; biochemical examination: total bilirubin ≤1.5 × upper limit of normal range (ULN), alanine aminotransferase (ALT) and aspartate aminotransferase (AST) ≤2× ULN, if liver metastasis, ALT and AST ≤5× ULN, endogenous creatinine clearance rate ≥60 mL/min; cardiopulmonary function is basically normal.

### 2.4 Exclusion criteria

Patients meeting the following criteria will be excluded: patients with severe heart, liver, or kidney damage or abnormal bone marrow function; past or concurrent with other malignant tumors, except for cured skin basal cell carcinoma and cervical carcinoma in situ; the general situation is poor, and those who cannot eat Chinese medicine; According to the judgment of the investigator, serious accompanying diseases that endanger the safety of the patient or affect the patient's completion of the study; pregnant or lactating women; people who are known to be allergic to the therapeutic drugs used in the trial.

### 2.5 Ethics approval

Ethics approval has been done by the Ethics Committee of the Affiliated Hospital of Guangdong Medical University (YJ2016-010KT-01). Information on any AE will be reported to the Ethics Committee a stable situation is reached. Moreover, the Ethics Committee has the duty to evaluate the progress of this study periodically.

### 2.6 Randomization and blinding

According to the random number generated by the computer, the drugs corresponding to the randomly assigned drug number are given in the order of participants’ enrollment. Participants who meet the eligibility criteria will be randomly assigned to the S-1 group and S-1 combined with XSLJZD group in a 1:1 ratio. Clinicians, patients, data collectors, and outcome assessors will be blinded to the group assignment. The allocation will be unblended if a severe adverse event (SAE) occurs and when the final data analysis is complete.

### 2.7 Interventions

In the S-1 group, S-1 was given orally twice daily at a dose of 80 mg/m^2^/d after a meal for 14 consecutive days, followed by 7 days of rest, and every 21 days counts as a cycle.

In the S-1 combined with XSLJZD group, S-1 was given orally twice daily at a dose of 80 mg/m^2^/d after a meal for 14 consecutive days, followed by 7 days of rest, and every 21 days counts as a cycle. According to the syndromes of the patients, XSLJZD was given combined treatment. The detailed composition of XSLJZD is as follows: Radix codonopsis 15 g, Rhizoma atractylodis macrocephalae 20 g, Poria 15 g, Pericarpium citri reticulatae 15 g, 10 g of Pinellia tuber, 10 g of Radix aucklandiae, 10 g of Fructus amomi, and 6 g of Radix glycyrrhizae.

### 2.8 Outcome measurements

#### 2.8.1 Primary endpoint

The primary endpoint is PFS, the time from randomization to disease progression (Evaluation of tumor progression about RECIST 1.1) or death.

#### 2.8.2 Secondary endpoints

The secondary endpoint is overall survival (OS) and Quality of Life Assessment (QOLA). OS is defined as the time from randomization to death for any reason. The last follow-up time is usually calculated as the time of death for the participants who have lost the visit before death (Additional file 1; Additional file 2; Additional file 3; Additional file 4).

QOLA, including symptom improvement, KPS, and AE assessment before and after treatment, was performed for 1 to 3 days before each cycle of treatment, Details are as follows:symptom improvement, mainly observe the changes of common symptoms of GC and CRC (pain, diarrhea, fatigue, poor acceptance, sleep), refer to the “Guiding Principles for Clinical Research of New Chinese Medicine” in the relevant TCM symptom integral quantification table of MDASI-TCM, compared to the scores accumulated for each symptom, analyze the difference before and after treatment^[30]^; KPS, comparative analysis before and after the difference; assessment of AE (safety outcomes). Assessment and grading of AE were based on the Common Terminology Criteria for Adverse Events (CTCAE) Version 4.0,^[31]^ AEs include nausea and vomiting, granulocytopenia, thrombocytopenia, anemia, impairment of liver and kidney function, diarrhea, peripheral neuritis, stomatitis, hand-foot syndrome, and any other new symptoms or diseases related to or unrelated to intervention. SAEs refer to the events that must be hospitalized or prolonged, permanently and seriously disabled, life-threatening, or death in clinical trials. SAE will be reported to the chief researcher and the hospital ethics committee, and the experiment will be stopped within 24 hours.

#### 2.8.3 Other measurements

Baseline was defined as pre chemotherapy (−7 to 0 days). During baseline examination, the patient's gender, age, marital status, education level, comorbidity, pathological type, tumor staging, radiotherapy and chemotherapy regimen, and medication records for the past 3 months will be recorded.

### 2.9 Follow-up

This study is expected to last for 5 years. Each patient will be followed until death or withdrawal from the study. Follow up frequency: follow up every 28 ± 3 days in the first year of the study, and record the PFS; then follow-up every 3 months and record the OS of 3 year and 5 year.

A detailed schedule of the study is presented in Table 1.

**Table 1:**
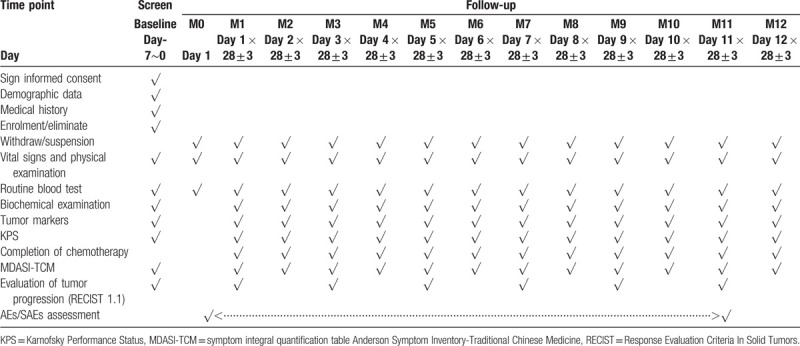
A detailed schedule of the trial.

### 2.10 Case elimination and withdrawal criteria

#### 2.10.1 Elimination criteria

The elimination criteria are as follows: case selection does not meet the inclusion criteria; it did not use the test drug; after the randomization, no data or major indicator missing, and the data is obviously incomplete; the drug prohibited by the test protocol was used, and the efficacy could not be evaluated.

#### 2.10.2 Withdrawal criteria

The withdrawal criteria are as follows: participants voluntarily request to withdraw from the test; SAE closely related to drugs occur, and toxicity intolerance makes the test impossible to continue; no reason for delaying treatment for >2 weeks; it cannot be treated according to the program, poor compliance; considering the interests of the participants, the researchers believe that the best choice is to terminate the study treatment.

#### 2.10.3 Solution

If the participant does not appear as planned during the follow-up period, the researcher shall know the reason as much as possible, record the reason on the case report form (CRF), contact the participant as much as possible to ask the participant to conduct a final visit, record the last medication time, try to complete the effectiveness and safety inspection when withdrawing from the study as specified in the scheme, and complete the safety follow-up period. AE and outcomes were fully recorded. According to the actual situation of the participants, researchers can suggest or provide new or alternative treatment methods to the participants.

If the participant is asking for termination of the study, researchers will retain the data and must follow-up in the specific follow-up steps specified in the study. If the participant refuses to visit further, he or she should continue to track his or her survival status unless the participant refuses to disclose further information or is contacted. In this case, no further research evaluations should be conducted, and no further information should be collected. The study sponsor may retain and continue to use all the information before the withdrawal of the participant's informed consent, unless the participant requests that the collected information be withdrawn.

### 2.11 Data collection

According to the original observation record of the study, the researcher will fill in the CRF in time, complete, correct, and check it. After checking, the data manager will input the original case report form into the online database repeated, and the database is password-protected. After importing the collected patient data into the database, the data administrator and the main researcher will perform a secondary check on the data, correct all the errors, and save them properly. When the experimental data are collected, the person leading the data analysis will analyze the data.

### 2.12 Sample size estimate

The RCT is an advantage test that uses an appropriate formula to estimate the sample size. Based on the results of our preliminary observational study and expert advice, we assume a significant level of α = 0.05, test efficacy (1-β) = 80%, and a pooled standard deviation of 9.59. Considering 10% dropout rate, the total sample size is expected to be 256 (128 of each carcinoma type, 64 in each group).

### 2.13 Statistical analysis

Analysis for primary endpoint PFS and secondary endpoint OS: the median of PFS and OS in each group was counted. Survival curves were plotted using Log-Rank test and Kaplan–Meier survival analysis.

Analysis for Symptom Improvement Assessment: behavioral status scores before and after chemotherapy (KPS), repeated measure's analysis of variance before and after treatment in the group; TCM symptom scores before and after chemotherapy (MDASI-TCM), paired *t* test before and after treatment in the group. The *t* test was used for comparison between groups.

All these statistical analyses were performed with SPSS 23.0 (SPSS Inc, Chicago, IL). A 2-tailed *P* value <.05 was considered statistically significant.

## 3 Protocol monitoring

An independent Response Evaluation Committee (IREC) was in place throughout the trial to monitored the study, which consists of 2 oncology imaging diagnostic and evaluation experts and one oncology clinical expert. Under the premise of blindness, the IREC carries out an independent third-party evaluation of the objective imaging data form all participants. The final was based on the evaluation of IREC. The participants of the clinical study will be informed that there will be relevant personnel to check during the study, but the privacy and data of the patients will be exactly protected. During the clinical study, clinical inspectors will conduct regular on-site monitoring visits to ensure that all content of the research protocol is strictly observed, and the original data are checked to ensure consistency with the CRF.

## 4 Data confidentiality

All records relating to the identity of the participant are kept confidential and the information will not be disclosed to the public beyond the limits permitted by relevant laws and/or regulations. The name of the participant will not be provided to the sponsor. Only the abbreviation of participant number and name is recorded in the medical record report form. If the participant's name appears in any other document, it must be hidden before a copy of the document can be provided to the sponsor. Research reports stored by computer must comply with local laws on data protection. Patients will be informed in writing that representatives of the study sponsor, ethics committee, or drug administration may review their medical records to check the collected information, and all individual information involved in the review will be strictly confidential and comply with localized data protection laws. If the results of the study are published, the personal identity of the patient will remain confidential. The researchers will keep a list checking the patient's records.

## 5 Discussion

According to the study of stage III or IV GC and CRC, maintenance therapy may make its survival benefit. S-1, a new oral fluorouracil chemotherapeutic drug, has been used more and more in the clinic after first-line chemotherapy for stage III or IV GC and CRC because of its longer drug concentration and lower side-effects.

In China, TCM is a common means to treat various diseases, including cancer. According to the current treatment guidelines for stage III or IV GC and CRC, chemotherapy is still the primary choice. The corresponding adverse reactions after chemotherapy, such as nausea and vomiting, have seriously reduced the quality of life of patients. Chemotherapeutic drugs and TCM have their own obvious advantages in cancer treatment. However, there are no more reports on the application of this combined model in maintenance therapy of stage III or IV GC and CRC.

Although chemotherapy combined with targeted therapy has a good effect, it inevitably kills normal cells, toxic accumulation, risk of drug resistance, and is limited to specific populations of genetic phenotypes, costly to maintain. The S-1 combined with TCM is convenient to take, combined to strengthen the body to eliminate pathogens, has fewer adverse reactions, used only in outpatient treatment, does not need for hospitalization, has relatively low price, affordable or patients, follows the current economic conditions of most patients in China. Therefore, this model can become an ideal choice for maintenance therapy of advanced GC and CRC in our country.

Based on the above consideration, we designed the study of XSLJZD combined with S-1 in the maintenance therapy of stage III or IV GC and CRC. It is an open, single-center, and randomized trial, which aims to determine the efficacy and safety of S-1 combined with XSLJZD in the maintenance therapy of stage III or IV GC and CRC. In addition, this study also provides meaningful clinical information for this combination model of chemotherapeutic drugs and TCM, laying the foundation for larger clinical trials in the future. However, the study has several limitations. First, this is a single-center trial, which is more difficult to recruit than multicenter trials. Therefore, it will take longer to recruit if enough samples are recruited. Second, although TCM formulas have been widely used in clinical practice in China, RCT with a well-designed and long-term follow-up is still lacking. Consequently, the results of the RCT will provide experience and evidence for developing guidelines and policies for facilitating the reasonable use of TCM formulas. We also hope that this research will open a new direction for the treatment of patients with stage III or IV GC and CRC.

## Acknowledgments

The authors thank the Affiliated Hospital of Guangdong Medical University for providing a platform and instruments for this experimental study.

## Author contributions

QLL and XCH designed the study and drafted the manuscript; XCH, QLL, XBL, KHH, HXY are responsible for the conduct of the study. WTO and HJZ conceived and designed the study, developed the statistical analysis plan, reviewed the manuscript. All authors approved the final version of the manuscript.

## Supplementary Material

Supplemental Digital Content

## Supplementary Material

Supplemental Digital Content

## Supplementary Material

Supplemental Digital Content

## Supplementary Material

Supplemental Digital Content
