# Supplementary material for: Clinical study of XiangShaLiuJunZi decoction combined with S-1 as maintenance therapy for stage III or IV gastric carcinoma and colorectal carcinoma
Source: Medicine (Baltimore). 2020 May 8;99(19):e20081. doi: 10.1097/MD.0000000000020081 (PMC7440293; doi:10.1097/MD.0000000000020081)
Supplement: Supplemental Digital Content [file medi-99-e20081-s001.pdf]

Ethical Approval 1

AF32 伦理审查批件

批件号	PJ2016004XY	受理编号	2016-010KT
项目名称	香砂六君子汤加减联合替吉奥维持治疗Ⅲ、Ⅳ期胃癌及大肠癌的临床研究		
试验药品名称	无	临床研究分期	NA
申办方	无		
合同研究组织	无		
研究单位	肿瘤中心		
主要研究者	梁启廉		
审查方式	√会议审查 ☐快速审查		
审查类别	初审后复审	会议审查日期	2016 年 4 月 1 日
审查委员	见《会议签到表》		
审查文件	1、复审申请<br>2、 知情同意书（版本号：2.0，版本日期：2016 年 3 月 8 日）<br>3、有关质疑答复		
批准文件	复审批准文件：<br>1、 复审申请<br>2、 知情同意书（版本号：2.0，版本日期：2016 年 3 月 8 日）<br>初始审查批准文件：<br>1、临床研究方案（版本号：01，版本日期：2016-01-25）		

审查意见

根据卫生部《涉及人的生物医学研究伦理审查办法（试行）》(2007)、SFDA《药物临床试验质量管理规范（2003)》、《医疗器械临床试验规定（2004)》、WMA《赫尔辛基宣言》和 CIOMS 《人体生物医学研究国际道德指南》的伦理原则，经本伦理委员会审查，同意按所批准的临床研究方案、知情同意书、招募材料开展本项研究。

请遵循 GCP 原则、遵循伦理委员会批准的方案开展临床研究，保护受试者的健康与权利。

研究开始前，请申请人完成临床试验注册。

研究过程中若变更主要研究者，对临床研究方案、知情同意书、招募材料等的任何修改，请申请人提交修正案审查申请。

发生严重不良事件，请申请人在获知后 24 小时内提交严重不良事件报告。

请按照伦理委员会规定的年度/定期跟踪审查频率，申请人在截止日期前 1 个月提交研究进展报告；申办者应当向组长单位伦理委员会提交各中心研究进展

地址：广东省湛江市人民大道南 57 号 广东医科大学附属医院 电话：0759-2386971 Email: fyllwyh@126.com

Ethical Approval 2

伦理审查意见

受理号	2016-010KT-01	意见号	YJ2016-010KT-01
项目名称	香砂六君子汤加减联合替吉奥维持治疗III、IV期胃癌及大肠癌的临床研究		
项目来源	广东医学院附属医院		
研究单位	广东医科大学附属医院		
主要研究者	梁启廉		
审查类别	年度/定期跟踪审查	审查方式	会议审查
审查日期	2018 年 05 月 25 日	审查地点	行政楼 1 号会议室
审查委员	见《会议签到表》		
审查文件	1. 研究进展报告		
批准文件	1. 研究进展报告		

审查意见

根据卫生部《涉及人的生物医学研究伦理审查办法》(2016)、CFDA《药物临床试验质量管理规范(2003)》、《药物临床试验伦理审查工作指导原则》(2010年)、《医疗器械临床试验质量管理规范(2016)》、《人类遗传资源管理暂行办法(1998)》、《人类遗传资源采集、收集、买卖、出口、出境审批行政许可事项服务指南(2015)》、WMA《赫尔辛基宣言》和 CIOMS 《人体生物医学研究国际道德指南》的伦理原则，经本伦理委员会审查，意见如下:

1、审查结果为：批准研究继续进行。

2、定期跟踪审查频率不变，同意延长批件有效期至 2019 年 12 月 31 日。

调整年度/定期跟踪审查频率	12 个月
下次跟踪审查截止日期	2019 年 04 月 03 日
批件有效期	2016 年 04 月 05 日至 2019 年 12 月 31 日
主任委员签字	[signature]
伦理委员会	广东医科大学附属医院机构审查伦理委员会
伦理委员会 GCP 声明<br>我院伦理委员组成及操作方式严格遵循 GCP（包括 ICH-GCP）及相关法律、法规的规定，实施各项操作规程。	
日期	2018 年 05 月 28 日

地址：广东省湛江市人民大道南 57 号广东医科大学附属医院电话：0759-2386971 Email: fyllwyh@126.com

Translation：Ethical Approval 1

Affiliated Hospital of Guangdong Medical University Institutional Ethics Committee

AF32 Approved ethical documents

**Approval number**	**PJ2016004XY**	**Acceptance number**	**2016-010KT**
**Project name**	**Clinical Study of XiangShaLiuJunZi Decotion Combined with S-1 as maintenance therapy for stage III or IV Gastric Carcinoma and Colorectal Carcinoma**		
**Test drug name**	**NO**	**Clinical study stage**	**NA**
**Sponsor**	**NO**		
**Cooperative research organization**	**NO**		
**Research unit**	**Cancer Center of the Affiliated Hospital of Guangdong Medical University**		
**Main researchers**	**Qi-Lian Liang**		
**Mode of review**	**Conference Review**		
**Review category**	**Review after the first trial**	**Date of review**	**April 1, 2016**
**Review committee**	**See the conference check-in form for details**		
**Review document**	**1. Review application**<br>**2. Informed consent form (version number: 2.0, version date: March 8, 2016)**<br>**3. Relevant questions and answers**		
**Approval document**	**Review and approval documents:**<br>**1. Review application**<br>**2. Informed consent form (version number: 2.0, version date: March 8, 2016)**<br>**Initial approval document:**<br>**1. Clinical research program (version number: 1.0, version date: January 25, 2016)**		

Review opinion

According to the Ministry of Health, "Ethical Review of Biomedical Research in Persons (Trial Implementation)" (2007), CFDA "Quality Management Regulations for Drug Clinical Trials (2013)", "Clinical Test Regulations for Medical Devices (2004)", WMA "Helsinki Declaration" And the ethical principles of the CIOMS International Code of Ethics for Human Biomedical Research, reviewed by the Ethics Committee, agreed to conduct this study approved clinical research protocols, informed consent, and recruitment materials.

Follow the GCP principles and follow the clinical protocols approved by the Ethics Committee to conduct clinical research to protect the health and rights of the subjects.

Applicants are required to complete a clinical trial registration before the clinical study begins.

If the main investigator is changed during the research process, any changes to the clinical research plan, informed consent, recruitment materials, etc., the applicant is requested to submit an amendment review application.

Serious adverse events occurred and applicants are required to submit a report of serious adverse events within 24 hours of informed.

Please follow the annual/regular tracking frequency set by the Ethics Committee and submit the research progress report one month before the deadline.

Translation：Ethical Approval 2

Affiliated Hospital of Guangdong Medical University Institutional Ethics Committee

Ethical review

**Approval number**	**2016-010KT-01**	**Acceptance number**	**YJ2016-010KT-01**
**Project name**	**Clinical Study of XiangShaLiuJunZi Decotion Combined with S-1 as maintenance therapy for stage III or IV Gastric Carcinoma and Colorectal Carcinoma**		
**Project source**	**Affiliated Hospital of Guangdong Medical University**		
**Research unit**	**Cancer Center of the Affiliated Hospital of Guangdong Medical University**		
**Main researchers**	**Qi-Lian Liang**		
**Review category**	**Annual/regular tracking review**	**Mode of review**	**Conference Review**
**Date of review**	**May 25, 2018**	**Review location**	**Administrative Building No. 1 Conference Hall**
**Review committee**	**See the conference check-in form for details**		
**Review document**	**Research progress report**		
**Approval document**	**Research progress report**		

Review opinion

According to the Ministry of Health, "Ethical Review of Biomedical Research in Persons" (2016), CFDA "Quality Management Standards for Drug Clinical Trials (2013)", "Guidelines for Ethical Examination of Drug Clinical Trials" (2010), "Clinical Tests for Medical Devices" Quality Management Regulations (2016), Interim Measures for the Management of Human Genetic Resources (1998), Guidelines for the Collection, Collection, Inheritance, Sale, Export, and Exit of Administrative Authorities for Human Genetic Resources (2015), WMA Helsinki Declaration The ethical principles of CIOMS and the International Ethics Guide for Human Biomedical Research are reviewed by the Ethics Committee and are as follows:

1. The result of the review is: approval of the study continues.

2. Regularly follow the frequency of review and agree to extend the validity of the approval to December 31,2019.

**Adjust the annual/regular tracking frequency**	**12 months**
**Next tracking review deadline**	**April 3, 2019**
**Approval period**	**April 3, 2019 to December 31, 2019**
**Signature of the chairman**	**Signature**
**Ethics committee**	**Ethics Committee of Affiliated Hospital of Guangdong Medical University**
**Ethics Committee GCP Statement**<br>**The composition and operation methods of our ethics committee strictly follow the GCP (including ICH-GCP) and related laws and regulations, and implement various operational procedures.**	
**Date**	**May 28, 2018**
