# Supplementary material for: Clinical study of XiangShaLiuJunZi decoction combined with S-1 as maintenance therapy for stage III or IV gastric carcinoma and colorectal carcinoma
Source: Medicine (Baltimore). 2020 May 8;99(19):e20081. doi: 10.1097/MD.0000000000020081 (PMC7440293; doi:10.1097/MD.0000000000020081)
Supplement: Supplemental Digital Content [file medi-99-e20081-s002.pdf]

湛江市科学技术局文件

湛科〔2018〕160号

关于下达2018年度湛江市科技发展专项资金竞争性分配项目的通知

各有关单位:

现将2018年度湛江市科技发展专项资金竞争性分配项目下达给你们，并就有关事项通知如下:

一、本次下达科技计划项目63项，经费1230万元，其中“海洋科技产业创新中心科技资源集聚工程专题”2018年度支持经费100万元/项，视承担单位2019、2020年的在湛江市海洋科技产业创新中心的基础设施、科研仪器和设备等投入情况和项目实施进度情况，总支持经费不超过400万元/项。

二、各县（市、区）科技主管部门应履行项目的日常监管职责，负责督促辖区的项目承担单位做好项目实施，并配合市有关部门组织开展项目的监督检查、绩效评价、验收结题和项目审计等工作。

三、各项目承担单位要抓紧组织实施项目，严格按照科技经费的使用范围和有关规定管好用好财政资金，按合同规定落实项目的配套经费，确保按期完成科研任务，提升创新能力。项目实施过程中，承担单位须按照要求填报上年度执行情况报告。项目完成后，要依照规定进行验收结题。

四、各项目承担单位须尽快登录湛江市科技业务管理阳光政务平台填报“湛江市科技计划项目合同书”，并抓紧办理项目资金申请：市直单位填报《预算单位专项资金使用申请表》，县（市、区）单位联系辖区科技主管部门及财政局办理。

纸质合同书请于12月14日前一式4份报送至湛江市生产力促进中心。联系人：庞燕，联系电话：3205352。

附件：《2018年度湛江市科技发展专项资金竞争性分配项目安排计划表》

湛江市科学技术局

2018年12月6日

附件：

2018年度湛江市科技发展专项资金竞争性分配项目安排计划表

序号	项目编号	项目名称	承担单位	金额（万元）
1、海洋科技产业创新中心科技资源集聚工程专题（专题编号：2018A201）				
1	2018A01010	湛江市恒兴海洋食品研发中心建设	广东恒兴集团有限公司	100
2	2018A01011	现代海洋生物科技创新中心建设及行业共性关键技术攻关	湛江科创海洋科技研究院有限公司	100
3	2018A01012	南方海参白沙参种业技术创新与应用	广东天海参威科技开发有限公司	100
**备注：**该专题2018年度支持经费100万元/项，视承担单位2019、2020年的在湛江市海洋科技产业创新中心的基础设施、科研仪器和设备等投入情况和项目实施进度情况，总支持经费不超过400万元/项。				
2、产学研协同创新专题（专题编号：2018A202）				
1	2018A01013	南美白对虾“兴海1号”新品种继代培育和产业化应用	湛江市国兴水产科技有限公司	40
2	2018A01014	优质杂交稻育繁推产学研协同创新产业化工程	广东天弘种业有限公司	40
3	2018A01015	优质菠萝果酒低温全汁发酵关键技术研究及应用	广东南派食品有限公司	40
4	2018A01016	海洋低聚寡糖生产关键技术研究及其产业化	湛江市博泰生物化工科技实业有限公司	40
5	2018A01017	分子鉴定技术和近红外光谱技术在饲料蛋白质量精准评价中的应用研究	湛江恒兴特种饲料有限公司	40
6	2018A01018	虾加工废弃物高效水解酶的克隆表达、特性及其应用	湛江市亨爱食品有限公司	40
7	2018A01019	面向集约化深水网箱养殖的多功能综合养殖作业平台研发与产业化	湛江渔宝现代渔业科技有限公司	40
8	2018A01020	适宜机械化的甘蔗新品种筛选与示范推广	湛江市金丰农业技术开发有限公司	40
3、工业技术攻关专题（专题编号：2018A203）				
1	2018A02009	多功能微细加工机床关键技术研究及产业化应用	岭南师范学院	25
2	2018A02010	南海环境风电设备钢构件涂层体系耐腐蚀性电化学快速评价技术研究及示范推广	岭南师范学院	25
3	2018A02011	锂离子电池用石墨烯包覆改性天然石墨负极材料的关键技术研究	广东海洋大学	25
4	2018A02012	新型电饭锅智能开发测试平台的研发	岭南师范学院	25

序号	项目编号	项目名称	承担单位	金额（万元）
5	2018A02013	海水淡化与制盐一体化技术中脉动能利用方法及装置的研发	广东海洋大学	25
6	2018A02014	高通量海洋微塑料分析系统研发	广东海洋大学	25
7	2018A02015	虾蟹加工副产物高值化利用关键技术研发及应用	中国热带农业科学院农产品加工研究所	25
8	2018A02016	神经根型颈椎病仿手法治疗机械装备及关键技术研究	岭南师范学院	25
9	2018A02017	贝壳粉基环保型涂料制备关键技术研究	广东海洋大学	25
10	2018A02018	麒麟菜来源多肽为有效成分研制防治特发性肺纤维化口服液	广东医科大学	25
11	2018A02019	蓄能高效冷热装置的研究及在饮料机产业化的应用	广东海洋大学	25
12	2018A02020	新型溶胶结合纤维保温材料的研发	湛江市红鹰铭德新材料科技有限公司	25
**4、高新技术企业树标提质行动计划专题（专题编号：2018A204）**				
1	2018A02021	数码快充电池用天然石墨负极材料制备的关键技术研究	湛江市聚鑫新能源有限公司	30
2	2018A02022	低蛋白无抗小猪配合饲料的开发与产业化示范	广东恒兴饲料实业股份有限公司	30
3	2018A02023	羽绒加工智能化除黑头技术的研发及产业化应用	湛江紫荆羽绒制品有限公司	30
4	2018A02024	基于电饭锅树标提质关键工艺研究与应用	广东威王集团有限公司	30
5	2018A02025	即食海蜇二次漂洗技术的创新	吴川市天然食品加工有限公司	30
6	2018A02026	基于热镀铝锌合金复合新材料技术的高防护性智能安全门及其产业化开发	广东恒中门业有限公司	30
**5、农业科技创新平台建设专题（专题编号：2018A205）**				
1	2018A03032	广东省热带作物装备科技创新中心建设——种茎类作物切种技术与装备研究	中国热带农业科学院农业机械研究所	10
2	2018A03033	茗禾现代农业科技创新中心（基地）	湛江市茗禾茶业有限公司	10
3	2018A03034	天然海蜇加工农业科技创新中心	吴川市天然食品加工有限公司	10
4	2018A03035	遂溪县电子商务产业园星创天地	湛江市启程电子商务有限公司	10
**6、医药应用基础和疾病防治技术研究专题（专题编号：2018A206）**				
1	2018A01021	小血管性认知障碍患者肠道菌群变化及通过影响小鼠小肠粘膜miR-146a而影响认知功能的机制研究	广东医科大学附属医院	3

序号	项目编号	项目名称	承担单位	金额（万元）
2	2018A01022	Hedgehog信号通路在非小细胞肺癌A549细胞放疗敏感性中的作用机制研究	广东医科大学附属医院	3
3	2018A01023	温敏水凝胶递送红景天苷对急性心肌梗死的治疗作用及机制研究	广东医科大学附属医院	3
4	2018A01024	脑缺血损伤后的血清外泌体对小胶质细胞活化的作用研究	广东医科大学	3
5	2018A01025	miRNAs通过调控长链非编码RNA-MALAT1介导三阴乳腺癌的侵袭和转移信号通路的实验研究	广东医科大学附属医院	3
6	2018A01026	基于竞争风险模型的鼻咽癌个体化可视化多临床结局风险预测模型研究	广东医科大学附属医院	3
7	2018A01027	炎症性肠病患者饮食营养、饮食行为调查及微量元素评估	广东医科大学附属医院	3
8	2018A01028	香砂六君子汤加减联合替吉奥维持治疗III、IV期胃癌及大肠癌的研究	广东医科大学附属医院	3
9	2018A01029	SIRT1介导氧化应激调控冠状动脉内皮缺血再灌注损伤的机制及干预作用研究	广东医科大学	3
10	2018A01030	含盐酸小檗碱四联疗法根除幽门螺旋杆菌的临床研究	湛江中心人民医院	3
11	2018A01031	骨质疏松骨折模型微结构的自动化评估基础研究	广东医科大学附属医院	3
12	2018A01032	新型生物敷料的研制及其用于糖尿病大鼠皮肤创面修复的研究	广东医科大学	3
13	2018A01033	3D打印辅助个性化手术治疗桡骨极远端骨折临床应用研究	湛江中心人民医院	3
14	2018A01034	探讨积雪草酸治疗顺铂所致急慢性肾损伤的作用及机制	广东医科大学附属医院	3
15	2018A01035	右美托咪定通过HMGB1/NF－ΚB抑制肠缺血再灌注诱导的自噬性细胞凋亡	广东医科大学附属医院	3
16	2018A01036	3D打印辅助膝关节单髁置换优化截骨设计及生物力学研究	广东医科大学附属医院	3
17	2018A01037	HIF2α拮抗剂对不完全射频消融后肝癌细胞增殖及转移影响的研究	广东医科大学附属医院	3
18	2018A01038	ADSCs治疗增生性瘢痕的分子生物学机制研究	广东医科大学	3
19	2018A01039	护士主导的吞咽管理模式对老年患者安全进食的影响研究	广东医科大学	3
20	2018A01040	抗衰老药二甲双胍对狼疮性肾炎小鼠的治疗作用及机制	广东医科大学附属医院	3
21	2018A01041	从湛江红树林内生真菌中分离的细胞松弛素H抗肺癌血管生成潜在靶点HIF-1α的研究	广东医科大学	3
22	2018A01042	基于天然产物蒽醌的组合化学合成与抗菌活性筛选	广东海洋大学	3
23	2018A01043	新型壳聚糖-熊果酸缀合物纳米纤维的构建及其在糖尿病足溃疡 中的应用	广东海洋大学	3

序号	项目编号	项目名称	承担单位	金额（万元）
24	2018A01044	SHV-12型超广谱β-内酰胺酶的定位对阴沟肠杆菌适应性的影响研究	广东医科大学	3
25	2018A01045	DPA预防和缓解老年痴呆的作用及机制研究	广东海洋大学	3
26	2018A01046	基于轴突再生的抗阿尔茨海默病药物太子参的物质基础、作用机制及其应用的研究	广东海洋大学	3
27	2018A01047	超声新技术对颈部淋巴结良恶性病变诊断价值的研究	广东医科大学附属医院	3
28	2018A01048	以TAP73的代谢调控为切入点探讨子宫内膜癌发病的分子机制	广东医科大学附属医院	3
29	2018A01049	虫草素通过MAPK/ERK 信号通路诱导不同类型人乳腺癌细胞凋亡的机制研究	广东医科大学附属医院	3
30	2018A01050	基于双眼交互式虚拟现实技术对儿童青少年近视患者注视稳定性及精细立体视的观察研究	湛江中心人民医院	3
