# Supplementary material for: Clinical study of XiangShaLiuJunZi decoction combined with S-1 as maintenance therapy for stage III or IV gastric carcinoma and colorectal carcinoma
Source: Medicine (Baltimore). 2020 May 8;99(19):e20081. doi: 10.1097/MD.0000000000020081 (PMC7440293; doi:10.1097/MD.0000000000020081)
Supplement: Supplemental Digital Content [file medi-99-e20081-s003.pdf]

广东医科大学附属医院文件

广东医附政发〔2018〕70号

关于公布2018年度院内资助类临床研究项目立项资助项目的通知

各科室、部门:

为加快推进我院临床研究的发展，提升我院临床研究水平，根据《关于印发<广东医科大学附属医院临床研究管理办法（试行）>的通知》（广东医附政发〔2016〕72号）的相关要求，本着公平、公正、公开的原则，经过项目申报、形式审查、专家评审、公开答辩及2018年10月30日党政联席会审议通过，同意对“Uncut Roux-en-Y”吻合与“Billroth II”吻合对远端胃癌患者腹腔镜D2根治术后远期并发症，生活质量影响的单中心、开放性、优效性随机对照研究”等7个项目（附件）予以立项资助。现予印发，请遵照执行。

特此通知。

附件：2018年度院内资助类临床研究项目立项资助名单

广东医科大学附属医院

2018年11月7日

附件

2018年度院内资助类临床研究项目
立项资助名单

姓名	项目名称	资助金额（万元/年）	资助年限（年）	课题编号
许庆文	Uncut Roux-en-Y吻合与Billroth II吻合对远端胃癌患者腹腔镜D2根治术后远期并发症，生活质量影响的单中心、开放性、优效性随机对照研究	10	4	LCYJ2018A001
谭建新	布地奈德/福莫特罗隔日使用与每日使用在儿童哮喘降级维持治疗阶段的疗效比较---多中心随机对照研究	10	5	LCYJ2018A002
刘　洲	粪菌移植治疗多发性硬化的优效性随机对照临床试验	10	5	LCYJ2018A003
陈　婷	羟氯喹治疗大量蛋白尿的狼疮性肾炎患者的疗效及安全性分析——单中心、开放性、随机对照临床研究	10	5	LCYJ2018A004
梁启廉	香砂六君子汤加减联合希罗达维持治疗Ⅲ-Ⅳ期结直肠癌的单中心、开放、随机对照的临床研究	10	5	LCYJ2018A005
陈嵘祎	雌二醇与女性无症状淋病相关性研究及诊断标准设定	10	5	LCYJ2018A006
梁　柱	单孔与三孔胸腔镜手术治疗I、II期非小细胞肺癌的临床随机对照研究	5	4	LCYJ2018B001
总计		65		

广东医科大学附属医院办公室　　　　2018 年 11 月 7 日印发

(共印 15 份)
