# Supplementary material for: Clinical study of XiangShaLiuJunZi decoction combined with S-1 as maintenance therapy for stage III or IV gastric carcinoma and colorectal carcinoma
Source: Medicine (Baltimore). 2020 May 8;99(19):e20081. doi: 10.1097/MD.0000000000020081 (PMC7440293; doi:10.1097/MD.0000000000020081)
Supplement: Supplemental Digital Content [file medi-99-e20081-s004.pdf]

Translation 1：Funding Documentation 1

Documents of Zhanjiang science and Technology Bureau

Zhanjiang science and Technology Bureau(2018)No.160

Notice on the science and technology development special fund competitive allocation project in Zhanjiang City ,China in 2018

All relevant units:

Now, we hereby give you the science and technology development special fund competitive allocation project of Zhanjiang City in 2018, and the relevant notice is as follows:

1. 63 projects of science and technology plan are issued this time, with a total funding of 12.3 million yuan, including one project of 1 million yuan supported by "science and technology resources gathering project of marine science and technology industry innovation center" in 2018, depending on the investment of infrastructure, scientific research instruments and equipment in Zhanjiang Marine Science and technology industry innovation center in 2019 and 2020 and the implementation progress of the project. The fund shall not exceed 4 million yuan for one project.

2. The project departments leading science and technology in each county shall perform the daily supervision responsibilities of the project, be responsible for urging the project undertaking units in the jurisdiction to do a good job in project implementation, and cooperate with the relevant municipal departments to organize and carry out the supervision and inspection, performance evaluation, acceptance conclusion and project audit of the project.

3. All project undertaking units shall promptly organize the implementation of the project, make good use of the financial funds in strict accordance with the scope of application and relevant regulations of the science and technology funds, implement the supporting funds of the project the provisions of the contract, ensure the completion of scientific research tasks punctual, and improve the innovation ability. During the implementation of the project, the undertaking unit must fill in and submit the performance report of the previous year as required. After the completion of the project, the acceptance shall be completed the regulations.

4. Each project undertaker must log in to Zhanjiang City Science and Technology Business Management Government Affairs Platform to fill in the "Zhanjiang Science and Technology Plan Project Contract" as soon as possible，and handle the project fund application as soon as possible: the units directly under the municipal government shall fill in the application form for the use of special funds of budget units, and the county units shall contact the science and technology competent department and the Financial Bureau of their jurisdiction for handling.

Please submit the paper contract in quadruplicate to Zhanjiang Productivity Promotion Center before December 14, contact person: Pang Yan, contact number: 3205352.

Attachment: competitive allocation project arrangement of special funds for science and technology development in Zhanjiang in 2018

Zhanjiang science and Technology Bureau
December 6, 2018

Attachment:

competitive allocation project arrangement of special funds for science and technology development in Zhanjiang in 2018

Serial number	Project number	Project name	Undertaking unit	Amount (ten thousand yuan)
1.Marine Science and Technology Industry Innovation Center Science and Technology Resource Agglomeration Project (Special Number: 2018A201)				
Omit				
2. Special Subject of Industry, Research and Research Association Innovation (Special Number: 2018A202)				
Omit				
3. Special Subject of Industrial Technology (Special Number: 2018A203)				
Omit				
4. Special Subject of Improve the quality of high-tech industries (Special Number: 2018A204)				
Omit				
5. Special Subject of Agricultural Science and Technology Innovation Platform Construction (Special Number: 2018A205)				
Omit				
6. Special Subject of Research on the basis of medicine application and disease prevention technology(Special Number: 2018A206)				
8	2018A01028	Clinical Study of XiangShaLiuJunZi Decotion Combined with S-1 as maintenance therapy for stage III or IV Gastric Carcinoma and Colorectal Carcinoma	Affiliated Hospital of Guangdong Medical University	3

Translation 2：Funding Documentation 2

Documents of Affiliated Hospital of Guangdong Medical University

<u>**Government affairs of Affiliated Hospital of Guangdong Medical University(2018) No. 70**</u>

Notice on the announcement of the project approval and funding of the hospital funded clinical research projects in 2018

Various departments and departments:

To speed up the development of clinical research in our hospital and improve the clinical research level of our hospital, according to the Notice on Printing and Distributing the Administrative Measures for Clinical Research of Affiliated Hospital of Guangdong Medical University (Trial) (Government affairs of Affiliated Hospital of Guangdong Medical University [2016] No. 72) Relevant requirements, the principles of fairness, justice and openness, after project declaration, formal review, expert review, public defense and the party and government joint meeting of October 30, 2018, agreed to "Uncut Roux-en-Y" The anastomosis and "Billroth II" anastomosis for remote gastric cancer patients with long-term complications after laparoscopic D2 radical surgery, a single-center, open-label, superior-effect randomized controlled trial of quality of life, and other seven projects (attached) were funded. It is now issued, please follow the instructions.

Hereby notify.

Attachment: List of funding support for clinical research projects funded by the hospital in 2018

Affiliated Hospital of Guangdong Medical University

November 7, 2018

Attachment:

List of funding support for clinical research projects funded by the hospital in 2018

Project responsible person	Project name	Funding amount (ten thousand yuan / year)	Year of funding	Project number
Omit				
Omit				
Omit				
Omit				
Qi-Lian Liang	Single-center, open, randomized controlled clinical study of Xiangsha Liujunzi Decoction combined with Xeloda in the maintenance therapy of stage III-IV Colorectal Carcinoma	10	5	LCYJ2018A005
Omit				
Omit				
Total		65		

Office of Affiliated Hospital of Guangdong Medical University Issued on November 7, 2018
